# Dose reduction and withdrawal strategy for TNF-inhibitors in psoriatic arthritis and axial spondyloarthritis: design of a pragmatic open-label, randomised, non-inferiority trial

**DOI:** 10.1186/s13063-019-4000-5

**Published:** 2020-01-15

**Authors:** Celia A. J. Michielsens, Nadine Boers, Nathan den Broeder, Mark H. Wenink, Aatke van der Maas, Elien A. M. Mahler, Michelle L. M. Mulder, Désirée van der Heijde, Frank H. J. van den Hoogen, Lise M. Verhoef, Alfons A. den Broeder

**Affiliations:** 1 Department of Rheumatology, Sint Maartenskliniek, Hengstdal 3, 6574 NA Nijmegen, The Netherlands; 2 Department of Rheumatic Diseases, Radboud Institute of Health Sciences, Radboud University Medical Centre, Nijmegen, The Netherlands; 3 Department of Rheumatology, Leiden University Medical Centre, Leiden, The Netherlands

**Keywords:** Spondyloarthritis, Psoriatic arthritis, Dose reduction, Tapering, Randomised controlled trial, TNF inhibitors

## Abstract

**Background:**

Tumour necrosis factor inhibitors (TNFi) are effective in the treatment of patients with spondyloarthritis (SpA), including psoriatic arthritis (PsA) and axial spondyloarthritis (axSpA). However, these drugs come with some disadvantages such as adverse events, practical burden for patients and high costs. Dose optimisation of TNFi after patients have reached low disease activity (LDA) has been shown feasible and safe in rheumatoid arthritis (RA). However, data on TNFi dose optimisation in PsA and axSpA are scarce, especially pragmatic, randomised strategy studies.

**Methods:**

We developed an investigator-driven, pragmatic, open-label, randomised, controlled, non-inferiority trial (DRESS-PS) to compare the effects of a disease activity-guided treat-to-target strategy with or without a tapering attempt in patients with SpA (PsA and axSpA combined), ≥ 16 years of age, who are being treated with TNFi, and have had at least 6 months of low disease activity. The primary outcome is the percentage of patients in LDA after 12 months of follow up. Patients are assessed at baseline, 3, 6, 9, and 12 months of follow up. Bayesian power analyses with a weakened prior based on a similar study performed in RA resulted in a sample size of 95 patients in total.

**Discussion:**

More knowledge on disease activity-guided treatment algorithms would contribute to better treatment choices and cost savings and potentially decrease the risk of side effects. In this article we elucidate some of our design choices on TNFi dose optimisation and its clinical and methodological consequences.

**Trial registration:**

Dutch Trial Register, NL6771. Registered on 27 November 2018 (CMO NL66181.091.18, 23 October 2018).

## Background

Tumour necrosis factor inhibiting agents (TNF-inhibitors, TNFi) are effective and safe in the treatment of spondyloarthritis (SpA) such as psoriatic arthritis (PsA) and axial spondyloarthritis (axSpA) [1, 2].

Treatment of SpA with TNFi is often done using a more informal treat-to-target (T2T) approach, than that is used in rheumatoid arthritis (RA) [3], setting a treatment target such as low disease activity (LDA), assessing disease activity and adjusting treatment if the treatment target is not reached. However, whether dose tapering or stopping once the treatment target has been reached is also part of the treatment strategy is controversial, as can be seen in the different recommendations and the American College of Rheumatology (ACR) (“we conditionally recommend against tapering of the biologic dose as a standard approach”) and Assessment of SpondyloArthritis International Society-European league Against Rheumatology (ASAS-EULAR) guideline (“tapering, but not stopping a bDMARD, can be considered in patients in sustained remission”). These differences reflect the lack of evidence supporting these statements [4, 5].

The question to taper or not is indeed important. Despite its effectiveness, the use of TNFi also incorporates downsides including high costs and adverse events such as injection site reactions, an increased risk of infections and practical burden for patients [6, 7]. Therefore, it makes sense to explore strategies that optimise the risk-benefit ratio of these drugs, such as dose optimisation after patients have reached LDA.

In RA, T2T dose optimisation has indeed been included in the European recommendations [8]. A recent Cochrane review showed that fixed dose reduction (e.g. by 50%) and disease activity-guided dose optimisation for TNFi is comparable to continuation of treatment, although discontinuation without reinstatement of TNFi in the case of a flare seems to be an inferior strategy [9]. Additionally, previous studies have demonstrated the strong cost-effectiveness of tapering in RA [10–12]. Compared to the considerable amount of evidence on dose optimisation for TNFi in RA, data on this subject in PsA and axSpA are scarce [3, 13].

In PsA, a systematic review summarised the limited current evidence on dose optimisation and withdrawal of biological disease-modifying anti-rheunatic drugs (bDMARDs) in PsA, and favoured dose optimisation over discontinuation because of the substantial risk of loss of remission [14]. So far, no randomised controlled trials have been performed on dose optimisation strategies in PsA.

In axSpA, two systematic reviews concluded that more or less comparable to PsA, TNFi reduction strategies are successful in sustaining clinical remission or LDA in about 50% of patients but that discontinuation often leads to flares [14, 15]. A few randomised controlled trials have been conducted on dose optimisation or discontinuation in axSpA, where similar results were shown as in RA with regard to maintaining remission by fixed dose reduction (e.g. by 50%) compared with full dose etanercept [16]. A randomised controlled non-inferiority trial reported by Gratacós et al. demonstrated non-inferiority between dose optimisation and full-dose TNFi treatment in patients with axSpA. However, TNFi treatment was reduced by 25% only, and the trial was lacking a sufficient disease activity-guided tapering algorithm [17]. A recent randomised controlled trial by Landewé et al. in patients with non-radiographic axSpA (nraxSpA) raised two important points. First, it showed that after 28 weeks of achieving inactive disease with adalimumab therapy, 47% of patients who discontinued adalimumab and switched to placebo had no flares during the following 40 weeks, supporting the possibility of (temporary) sustained remission after discontinuation of TNFi in patients with nraxSpA. Furthermore, the authors state that the majority of patients who did have a flare did fully recover to their previous state of clinical remission after adalimumab reinstatement. The question arises whether the placebo group would have fewer flares if adalimumab was tapered instead of abruptly stopped. Interestingly, predictors for maintaining drug-free remission were not identified [18].

Still, prospective and randomised controlled strategy studies of dose optimisation (including stopping) in both PsA and axSpA are absent. Summarising the evidence, it seems plausible that a relevant proportion of patients with PsA or axSpA can maintain LDA following dose optimisation strategies or discontinuation of their TNFi once remission or LDA is reached, but this strategy has not been put to the test in a strategy RCT. Of note, this subject has been prioritised in the research agenda of some of the large rheumatology associations [3, 5].

We therefore set out to perform a pragmatic, open-label, randomised, controlled, non-inferiority strategy trial comparing a TNFi T2T strategy with or without a tapering attempt. During the design of this trial, a number of choices were made that we would like to address in this paper.

## Methods

### Design

**Fig. 1:**
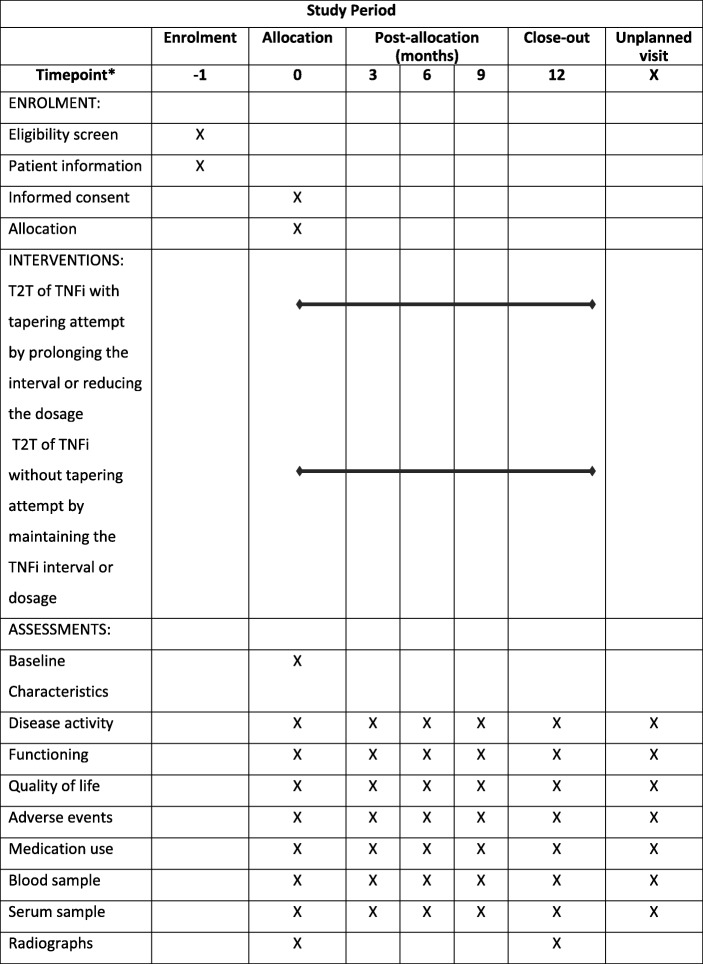
Standard protocol items: recommendation for interventional trials (SPIRIT) figure: trial visits and assessments. *Schedule of enrolment, time point in months. Patients visit the outpatient clinic every 3 months and if necessary extra visits are warranted. TNFi, tumour necrosis factor inhibitor

The dose reduction strategy study of TNF inhibitors in psoriatic arthritis and axial spondyloarthritis patients (DRESS-PS) is a pragmatic, open-label, mono-centre, randomised, controlled, non-inferiority strategy trial, and is currently recruiting patients at the departments of Rheumatology of the Sint Maartenskliniek in Nijmegen and Woerden and at the department of Rheumatic Diseases of the Radboud University Medical Centre (Radboudumc) in Nijmegen, the Netherlands (Fig. 1; Standard protocol items: recommendation for interventional trials (SPIRIT) checklist is shown in Additional file 1). The study design is inspired by the original DRESS study [19, 20].

### Objective

The primary objective is to investigate whether a protocolised T2T strategy of TNFi with a tapering attempt in patients with PsA and axSpA is non-inferior compared to T2T without a tapering attempt regarding the proportion of patients with LDA at 12 months using a pre-set non-inferiority margin of 20%. We chose having LDA at 12 months of follow up as our primary outcome. Persistent flare - the outcome used in the RA DRESS study - was not feasible as an outcome, since there are currently no validated flare criteria to appropriately detect persistent flares in PsA or axSpA, as opposed to RA (discussed later, p.17) [21]. Another option was to use the area under the curve (AUC) (mean time weighted) for disease activity. However, in our view this does not reflect the main outcome of a tapering strategy, because differences will probably occur between the two groups due to tapering-associated short-lived flares. While these short-lived flares are important, we assume that they can be treated easily, and have no sequelae like loss of function, reduced quality of life (QOL) or important radiological damage, and that being in LDA at the study end more truly reflects the main goal of trial and error tapering. Furthermore, a mean time weighted or an AUC value is difficult to interpret, and does not easily allow for combination of both diseases, since different disease activity measures are used. Our definition of LDA is based on the Psoriatic Arthritis Disease Activity Score (PASDAS) and Ankylosing Spondylitis Disease Activity Score (ASDAS) criteria, but also involves the absence of extra-axial manifestations, since treatment decisions in PsA and axSpA are not solely based on the PASDAS and ASDAS but also encompass skin and eye manifestations and inflammatory bowel disease, which are not sufficiently covered in the PASDAS and ASDAS alone.

Several secondary outcomes will be compared between the two groups, including disease activity, flares, function, quality of life, costs, co-medication and safety. Our main secondary objectives are to assess the proportion of patients in the intervention group that can successfully taper or discontinue TNFi use and the percentage defined daily dose (DDD) of TNFi use; to compare the change in the PASDAS and ASDAS at each time point; to compare the cumulative incidence and number of flares between the T2T groups with or without a tapering attempt; to compare the functioning in both groups (Health Assessment Questionnaire Disability Index (HAQ-DI) and Bath Ankylosing Spondylitis Functional Index (BASFI)); to compare the proportion of patients using non-steroidal anti-inflammatory drugs (NSAIDs), corticosteroid or conventional synthetic/biological/targeted synthetic disease-modifying anti-rheumatic drug (cs/b/tsDMARDs) in both groups; to compare the occurrence of adverse events, especially infections. In addition, we will determine the cost effectiveness of T2T with or without a tapering attempt and investigate whether baseline factors are able to predict successful dose optimisation.

### Patients

We have chosen to combine PsA and axSpA in this study mainly for feasibility reasons. Furthermore, PsA and axSpA share pathophysiological, genetic and clinical characteristics, and as current treatment options are almost identical with respect to type of drugs used, dosing and concomitant DMARDs used (Table 1) and finally because preliminary dose optimisation data are similar, we felt this was possible without too much risk of different effects in the two diseases. The T2T principle is also already widely pursued in multiple chronic inflammatory disorders, including PsA and axSpA, indicating that this overarching principle seems disease agnostic [9, 17, 18, 22]. Additionally, the outcome of non-inferiority of the tapering strategy is not dependent on the percentage of patients that can taper or stop, but on the implementation of the T2T strategy and the effectiveness of increased or restarted dosing on disease activity, and we do not anticipate effect modification between the two closely related diseases.

**Table 1:** Overview of DMARDs in psoriatic arthritis, radiographic axial spondyloarthritis and non-radiographic axial spondyloarthritis

	Psoriatic arthritis	Radiographic axial spondyloartritis	Non-radiographic axial spondyloarthritis
bDMARDs			
TNFi	Adalimumab *40 mg 1x/2 weeks Certolizumab *200 mg 1x/2 weeks Etanercept *25 mg 2x/week or 50 mg 1x/week Golimumab *50 mg 1x/month Infliximab *5 mg/kg 1x/8 weeks	Adalimumab *40 mg 1x/2 weeks Certolizumab *200 mg 1x/2 weeks Etanercept *25 mg 2x/week or 50 mg 1x/week Golimumab *50 mg 1x/month Infliximab *5 mg/kg 1x/8 weeks	Adalimumab *40 mg 1x/2 weeks Certolizumab *200 mg 1x/2 weeks Etanercept *25 mg 2x/week or 50 mg 1x/week Golimumab *50 mg 1x/month
Anti-IL-17	Secukinumab *150 mg 1x/month (up to 300 mg) Ixekizumab *80 mg 1x/4 weeks	Secukinumab *150 mg 1x/month	
Anti-IL-23, IL-12	Ustekinumab *45 mg 1x/12 weeks		
CTLA4-Ig	Abatacept *125 mg 1x/week		
PDE-4i	Apremilast *30 mg 2x/day		
JAKi	Tofacitinib *5 mg 2x/day		

*DMARD* disease-modifying anti-rheumatic drug, *bDMARD* biological DMARD, *TNFi* tumour necrosis factor inhibitor, *IL* interleukin, *CTLA4-Ig* cytotoxic T-lymphocyte associated protein 4-immunoglobulin, *PDE-4i* phosphodiesterase-4 inhibitor, *JAKi* janus kinase inhibitor

*Registered dosage in psoriatic arthritis, axial spondyloarthritis and non-radiographic axial spondyloarthritis

Patients with PsA or axSpA diagnosed clinically by the treating rheumatologist (and supported by Classification Criteria for Psoriatic Arthritis (CASPAR) and ASAS classification criteria) are included. To have optimal generalizability of our study to daily clinical practice, we pragmatically decided to keep the amount of inclusion and exclusion criteria as limited as possible. These patients are eligible if they have LDA up to 6 months prior to inclusion and are using > 50% of the authorized DDD of a TNFi (Table 2). Treatment decisions are made based on objective and subjective disease activity scores, by shared decision making between rheumatologist and patients. In patients with PsA, stable LDA is defined as having a Psoriatic Arthritis Disease Activity Score (PASDAS) ≤ 3.2 and body surface area involvement (modified BSA) ≤ 3%, used as a target by rheumatologists in routine practice and in the minimal disease activity (MDA). In axSpA this is defined as an Ankylosing Spondylitis Disease Activity Score (ASDAS) < 2.1 in PsA and axSpA an absence of active extra-axial disease-related symptoms caused by Crohn’s disease, ulcerative colitis, uveitis or psoriasis is also required for LDA, or when formal measurements are not available, the judgement of the physician and patient can be used instead. Patients with extra-axial manifestations of disease, such as inflammatory bowel disease (IBD), uveitis or psoriasis are included in our study, unless currently active extra-axial manifestations prevent dose optimisation. If extra-axial symptoms develop during treatment, physicians are allowed to treat them by delaying tapering or increasing TNFi dosage. We deemed exclusion of patients with inactive extra-axial manifestations unnecessary since in the psoriasis tapering trial patients seem to respond well to tapering strategies [23] and a similar study in patients suffering from IBD is currently being conducted [24].

**Table 2:** Dose optimisation strategy for TNFi

TNFi	100%	66%	50%	0%
Adalimumab/Certolizumab	40 mg 14 days interval	40 mg 21 days interval	40 mg 28 days interval	Stop TNFi
Etanercept	50 mg 7 days interval	50 mg 10 days interval	50 mg 14 days interval	Stop TNFi
Golimumab	50 mg 4 weeks interval	50 mg 6 weeks interval	50 mg 8 weeks interval	Stop TNFi
Infliximab	5 mg/kg 8 weeks interval 3 mg/kg 8 weeks interval	3 mg/kg 8 weeks interval 2.25 mg/kg 8 weeks interval	1.5 mg/kg 8 weeks interval 1.5 mg/kg 8 weeks interval	Stop TNFi

*TNFi* tumour necrosis factor inhibitor

We exclude only patients whose comorbidity could interfere with our protocolised dose optimisation strategy, thus being unable to participate due to the required treatment with TNFi (e.g. active Crohn’s disease, ulcerative colitis, uveitis, psoriasis), or when it is expected that the outcome cannot be measured (short life expectancy, planned major surgery). Pregnant women are also excluded from participation in this trial. A previous (successful or unsuccessful) attempt at dose optimisation is allowed if attempted more than 2 years ago. The use of concomitant NSAIDs and csDMARDs is allowed before and during participation in this study, though the intake of NSAIDs has to have been stable at least 8 weeks before inclusion.

We decided to include not only patients in remission, but extended this to patients with LDA 6 months prior to participation, for two reasons. First, this increases both the generalizability of the study and the feasibility, since many physicians and patients do not strive for remission, and there are no data on the diseases showing that a treatment target of remission results in better outcomes than a treatment target of LDA. Second, in studies with RA, there is no evidence that baseline disease activity is strongly associated with a chance of successful tapering [25], so this choice is not expected to jeopardize internal validity due to introducing effect modification.

### Ethical considerations

The study received ethical approval from the CMO region Arnhem Nijmegen (NL66181.091.18) and has been registered in the Dutch Trial Register (NTR 7640). Privacy of patients is protected according to Dutch law AVG (*Algemene Verordening Gegevensbescherming*), by using anonymized data and restricting access to patient identification logs. We established a Data Safety Monitoring Board (DSMB) where four independent DSMB members, of whom two are rheumatologists, one a pharmacist and one a biomedical scientist, will discuss and review data on recruitment, efficacy, safety, protocol adherence and protocol updates on Good Clinical Practice (GCP). The trial is investigator-driven, and funded by ReumaNederland (funding number 17–3-303).

### Patient recruitment

All eligible patients are selected and approached based on information from the electronic health record according to the aforementioned inclusion criteria. Patients are asked by their treating rheumatologist to join this study, by letter accompanied by the patient information sheet and the informed consent letter. Patients receive this information two weeks prior to a planned outpatient clinic visit. At the outpatient clinic visit (which is referred to as visit − 1), the study is discussed and informed consent is obtained.

### Randomisation and blinding

Participants are randomised using a computer-generated procedure, stratified by disease and for use of csDMARDs, to ensure equal distribution and prevent bias. We did not stratify by the different types of TNFi, since we expect a similar mechanism of action while tapering as demonstrated in previous studies [9]. Studies on dose optimisation with different types of TNFi, though mainly including data on adalimumab and etanercept, show no major difference in clinical outcome after dose optimisation or stopping. Patients are randomised in a ratio of 2:1 to the dose T2T strategy with or without a tapering attempt, respectively. The intervention group is larger to ensure that a more potential predictive factors for response can be studied in multivariate prediction modelling in the dose optimisation group. Randomisation blocks using variable block sizes (multiples of 3 or 6) are used to more closely achieve the intended allocation ratio and to prevent the allocation being predictable for the treating rheumatologist. Patients, physicians, nurses and researchers are not blinded during this study. Analyses are blinded to treatment allocation.

### Interventions

#### Control

Patients who are allocated to the control group continue their treatment by their treating rheumatologist based on a standardised treatment protocol. Patients visiting the outpatient clinic every three months are treated according to the T2T principle without a tapering attempt. Disease activity is measured at every visit, and we aim to achieve PASDAS ≤ 3.2 in patients with PsA and ASDAS < 2.1 in patients with axSpA. Extra visits are warranted if patients report complaints related to an increase in disease activity in between regular visits. The treating rheumatologist receives advice from the research physician at every visit, on what should be done according to the protocol. However, all treatment choices are left to the discretion of the treating rheumatologist in shared decision making with the patient. When patients experience a flare, in spite of the maximal TNFi dose, treatment is adapted accordingly by the rheumatologist to ensure optimal care. This might include adding NSAIDs, especially in axSpA, or use of glucocorticoids. Patients visit the outpatient clinic after 4 weeks to evaluate the effects of the treatment administered. In the case of a persistent flare, patients are switched to another TNFi or non-TNFi class of bDMARDs as per our treatment protocol. Since treatment changes are based on shared decision making between patient and physician in daily clinical practice, in patients experiencing flare and not meeting the proposed flare criteria, treatment can be intensified nevertheless. Dose optimisation or discontinuation for reasons other than adverse events is discouraged but allowed.

#### Intervention

The treatment in the intervention group is identical to the control group with the addition of a tapering attempt, leading to a trial and error dose-optimisation and eventually discontinuation. The interval of TNFi administration is extended gradually every 3 months in patients in the intervention group. Patients using the full dose discontinue the TNFi at approximately 6 months and are closely monitored for another 6 months, for a study total duration of 12 months. The dose optimisation steps from 100% to 66% and 50% before stopping for the different TNFi are depicted in Table 2. If a patient is using another dose regimen than the one proposed above, an alternative dose optimisation strategy is used. Patients who are not receiving full dosage of their TNFi step in at the nearest dosing interval. Patients encountering a flare are treated accordingly, following the same standardised protocol as the control group. All patients experiencing a flare are seen 4 weeks later. Should a flare persist, after receiving other medication, the dose is adjusted to the last effective interval or dosage. During this month patients do not continue dose optimisation but maintain the last effective interval or dosage. When the latter does not suffice to improve the disease activity, the patient is advised to switch to another bDMARD following the standardised treatment protocol.

#### Outcomes

As main disease activity measures in the study, we have chosen the PASDAS (for PsA) and ASDAS (for axSpA). Since a subset of patients with PsA and AxSpA have extra-axial manifestations of disease, we also assess the occurrence of these manifestations and patient and physician opinion on disease activity.

Several known disease-specific measures for PsA were reviewed and screened for reliability, validity and feasibility. In the process of selecting a suitable disease activity measurement tool for PsA in this trial, certain criteria had to be met in order to be feasible for use in routine clinical practice. Furthermore, the disease activity measure had to cover different domains of the disease and should be able to distinguish between different levels of disease activity, using clear cut-off points for disease activity and improvement.

PsA activity often includes pain or swelling of the ankles and distal interphalangeal joints in the hands, which makes Disease Activity Score 28-C-reactive protein (DAS28-CRP) a less suitable measurement tool, since it only measures 28 joints and does not include evaluation of these joints. The Disease Activity in PSoratic Arthritis (DAPSA) does not have a normal distribution and is not sensitive enough to change, having less favourable clinimetric properties compared to PASDAS [26]. A third option is the use of the minimal disease activity (MDA) criteria, as LDA as calculated by PASDAS is comparable with MDA. However, the MDA criteria result in a binominal score (yes/no), which provides less information than for example the PASDAS. Furthermore, since the MDA includes scoring of dermatological manifestations, it is less practical to use in daily clinical practice. Last, the Composite Psoriatic Disease Activity Index (CPDAI) seems to have less discriminative ability in distinguishing high and low disease activity than the other outcome measures [26–29]. Therefore, we opted to use PASDAS as a disease-specific activity measure for PsA, due to the following reasons: it is a continuous disease activity index that includes arthritis (66/68 joint score), dactylitis, enthesitis, physician disease activity visual analogue scale (VAS) score, patient disease activity VAS, patient-reported physical function and CRP [28]. To cover the dermatological component of PsA in our trial, psoriasis is measured by modified BSA (mBSA), since an increase in psoriasis activity is not sufficiently covered by the PASDAS. The mBSA ranges from 0 to 1 and 2 and correlates with no skin manifestations to ≤ 3% and > 3% of the body surface area covered.

In axSpA, the BASDAI and ASDAS are both acceptable disease activity measurement tools. We decided to use the ASDAS, since it also includes an objective measure with CRP [5] and has an established longitudinal relationship with structural damage, supporting its construct validity. Also, several cut-off values have been well-validated, including minimal clinically important worsening (MCIW).

As mentioned before, patients are frequently asked about the occurrence of active extra-axial disease manifestations such as Crohn’s disease, ulcerative colitis and uveitis.

#### Definition of flare

Since validated criteria for persistent flare are lacking, we consider patients to have a flare in several situations: when a patient experiences loss of LDA as defined by PASDAS > 3.2 and ASDAS ≥ 2.1, has an increase in disease activity (≥ 0.8 points of PASDAS for PsA or 0.9 for axSpA), has an important worsening of mBSA, or in the occurrence of active extra-axial symptoms as judged by the treating rheumatologist, with a duration longer than 1 month. Since treatment changes are based on shared decision making between patient and physician in daily clinical practice, if patients experience flare and do not meet the proposed flare criteria, treatment can be intensified nevertheless.

PsA flare is defined as an increase in PASDAS ≥ 0.8 points, since this increase equals the measurement error of the instrument [30]. Choosing 1–2 times the measurement error to define the MCIW is comparable to other instruments such as the DAS28-CRP used in RA and ASDAS in axSpA. In the dermatological domain, clear cut-off values for important worsening are lacking for mBSA and treatment is adjusted as judged by the treating rheumatologist and patient, since this is a very individual choice. AxSpA flare is defined as an increase in ASDAS ≥ 0.9 (the published MCIW for ASDAS) [31, 32]. We consider our flare criteria rather sensitive and therefore think we can detect any significant flare.

### Non-inferiority margin

The choice for a suitable non-inferiority (NI) margin is essential for a NI trial to provide clinically relevant conclusions [33, 34]. We chose a NI margin of 20%. In the DRESS study, 16% of patients were successfully stopped and 45% tapered, meaning 60% benefitted from a dose optimisation strategy, resulting in a number needed to treat (NNT) of about 1.5. If the confidence interval (CI) around the point estimate of the proportion of patients in LDA indeed remains below 20%, this strikes an acceptable balance between the advantages of dose optimisation such as reduced injection burden and risk of side effects, and the harm incurred from loss of LDA (with a substantially higher NNH of 5).

### Assay sensitivity

Since this is a non-inferiority trial, assay sensitivity, e.g. the ability of the trial to detect inferiority if it is present, is important to take into account. Assay sensitivity can be supported by a third arm in which a clearly inferior treatment is tested leading indeed to the conclusion of inferiority. In our study this would for example constitute an arm in which TNFi are not reinstated when flares occur. However, for several reasons we chose not to include a third arm. First, we use validated outcome measures (PASDAS and ASDAS) with known sensitivity to change, and several superiority studies have shown that these can detect differences between placebo and active treatment. Second, similar trials in RA and psoriasis have indeed proven inferiority of dose optimisation with regard to several outcomes, both primary and secondary. In the DRESS study inferiority was shown on short flares, minimal radiographic progression and AUC DAS28 [20], while STRASS was inferior on the DAS28 score at 18 months [35]. In psoriasis, a different disease entity, the CONDOR study showed inferiority on the PASI score [23]. Considering this, we feel that assay sensitivity of our trial design is sufficiently supported, and find it both unethical and impractical to randomise patients into an arm in which medication is not reinstated, as it would induce more patient harm, and require a substantially larger sample size.

### Sample size considerations and statistical analyses

We assume that both treatment arms have a prevalence of LDA state of 0.8 (80%) at 12 months, thus assuming 20% will lose LDA in both groups, as expected due to regression to the mean, based on previous research and clinical practice [18, 20]. We expect that dose optimisation will cause more (short-lived) flares, however we do not anticipate a permanent loss of LDA state after a T2T strategy with compared without a tapering attempt, because of the short longevity of the flares before reinstatement of the TNFi.

As motivated above, we take the non-inferiority margin of − 0.2 (20%) and the randomisation ratio of 2:1 of intervention versus control. The ratio of 2:1 for intervention and control sample size is chosen to be able to include more determinants in a prediction model for successful dose optimisation. The sample size of the study is based on a Bayesian analysis where non-inferiority will be claimed if the lower limit of the Bayesian 95% credibility interval of the difference is above − 20%. We chose a Bayesian sample size approach to be able to include information from the original DRESS study, resulting in a prior tending towards non-inferiority of dose optimisation [20]. This will enable us to take into account the previous evidence on TNFi tapering and reduces the required sample size without compromising the power of our study.

As we are only interested in claiming non-inferiority of a T2T strategy with versus without a tapering attempt, we only want to control the probability of claiming non-inferiority when inferiority is present. Taking into account the prior evidence, favouring non-inferiority of dose reduction, we choose to control this probability at 10% instead of 5% in line with the Food and Drug Administration (FDA) guidance on Bayesian statistics, which recommends a less stringent control of frequentist type I error if prior information is favourable [36]. In the RA DRESS study, a difference in persistent flare (which would lead to loss of LDA at 12 months) of − 2% was observed (90% LDA in 59 patients on continuation and 88% in 121 patients with dose optimisation). To make sure this prior based on the previous study does not have too much weight in the Bayesian analysis, since RA is a different disease, the precision of this prior is reduced and the prior is taken as normally distributed with mean − 2% and standard deviation of 22.5%. Each percentage is estimated from 500 simulated datasets of the control group having an LDA proportion of 80%. Bayesian analysis is performed with a generalized linear model with identity link and binomial distribution using a normally distributed prior with mean − 2% and standard deviation of 22.5%. We will recruit 90 subjects in total (30 vs 60) so as to have 80% power to claim inferiority when the true difference is 0%. Adding 5% dropout, the total number of patients needed is 95 patients. Although this is the minimum number of patients needed, we will attempt to include this number of patients for both PsA and axSpA separately, to achieve sufficient power for an analysis stratified by disease.

### Data analyses

Primary analyses will be per-protocol (PP), as this is the most conservative for a non-inferiority study. Additionally, analyses are performed on an intention-to-treat basis (ITT). For PP analysis, we include intervention patients that attempted at least one dose optimisation step and control patients who did not attempt dose optimisation for reason of treatment relaxation per se, but only when medically required such as in the case of adverse events or contraindications. Descriptive statistics are provided using mean and standard deviation (SD), median (p25-p75) or frequencies/percentages depending on the type of distribution of the data. For exclusion and dropout, numbers and reasons are reported to ensure internal validity. Missing values are imputed using multiple imputations when meeting the assumption of missing completely at random (MCAR)/missing at random (MAR), as imputation always increases precision and often also reduces bias [37, 38].

## Discussion

In addition to the motivations we provide for all the design choices we made in our study, we want to discuss some important overarching questions. We have decided to include patients with PsA and axSpA combined in one trial, as we argue that these disease entities are sufficiently similar and treated with similar TNFi to be analyzed in one composite group, thus also making the study more feasible. This leads to an interesting question: when are groups of patients similar or different enough to warrant separate studies? There are many examples that ask for a similar judgement call. In RA, for example, rheumatoid factor positive (RF+) and rheumatoid factor negative (RF-) patients are both included in the majority of studies, while pathophysiologically, they might be very different. Other examples of diseases studied that may have less in common than PsA and axSpA are abundant; granulomatosis with polyangiitis (GPA), with myeloperoxidase- (MPO) or proteinase 3- (PR 3), or anti-neutrophil cystoplasmic antibody (ANCA), pneumoniae with several causative agents, or studies on IBD including both Crohn’s disease and ulcerative colitis. Deciding to combine or split patients with different characteristics within a family of diseases remains a matter of specific judgement, especially whether considerable effect modification is to be expected on the primary outcome measure. Considering their similarities, for example, it could be argued that PsA and psoriasis treatment trials could be performed combining the diseases, but since these diseases are treated by different specialists and the treatment often showcases a different effect on skin and joints, this is not usually done. In this case, considering the feasibility, similarities in treatment, the existence of validated disease measurement scores, the possibility of tapering in a similar relevant proportion of patients and recovering to a previous state of clinical remission after reinstatement, patients are analysed in one group.

Another interesting point that can be disputed is whether these kind of T2T studies produce meaningful results. After all, diseases that can be measured, and treated with effective interventions should always be eligible for T2T strategies that ultimately will lead to results that are identical to any alternative targeted strategy. Flares or relapses of disease can after all be countered by (temporarily) increasing treatment dosages. This yields similar disease activity scores in the compared groups in the long run. Indeed, this is seen in many RA T2T studies in which all treatment arms converge after a certain amount of time to similar outcomes [39–43]. Proving non inferiority may therefore be a redundant goal, as per definition, this will always be achieved, yielding identical results in both groups. More evidently, if such a T2T study fails to prove non-inferiority, this must mean the execution of T2T was suboptimal, and this of course leads to circularity by definition. Of note, the prerequisite is that the T2T strategy study is adequately performed and sufficiently powered. The STRASS study, for example, failed to show non-inferiority in the T2T strategy due to insufficient recruitment [35]. However, the *proof of the pudding is in the eating*, and although logically sound, a strategy using T2T should be tested in a controlled trial, if only to demonstrate the feasibility of achieving such a level of treat-to-target.

One of the main goals of tapering strategies is to reduce healthcare costs. However, costs of TNFi have already lowered - at least in Europe - with the introduction of biosimilars, which results in price competition, as witnessed by the recent 80% + discounts on adalimumab offered by AbbVie in Europe and the Netherlands especially [44, 45]. As more outpatient visits are required during tapering and more time has to be invested in treating flares, it remains to be seen how the cost effectiveness of dose optimisation strategies evolves over time. However, costs associated with side effects like infections might also play a role, which could be a burden and cause work productivity loss. Also, besides cost reduction, benefits of dose optimisation include reduced patient burden due to self-injection and lower risks of adverse events. These benefits alone warrant the exploration of dose optimising strategies.

Moreover, given the benefits, the question arises as to who is responsible for the execution of dose optimisation research. Pharmaceutical companies often perform efficacy trials and are usually obliged to monitor information about the drugs’ safety and efficacy in so-called phase IV studies after authorization. However, when implementation of a certain treatment is established, reassessment of the efficacy in the case of dose optimisation or discontinuation is often neglected. Therefore, many dose optimisation studies are currently financed by public funds. When such trials *are* executed by pharmaceutical companies, the study designs tend to favour continuation of treatment, with the results following suit. For example, the choice not to follow a T2T treatment strategy (or at least reinstate therapy in the case of flare) and lack of cost-effectiveness analyses. Furthermore, it is expected that patients benefit from as little exposure to medication as possible following a dose-activity-guided strategy. Therefore, it could be debated whether it is the pharmaceutical company’s responsibility to conduct well-designed phase III/IV dose-reduction studies for authorized drugs, e.g. after several years of use. Recent developments in this field include that pharmaceutical companies receiving approval for the use of TNFi in nraxSpA are obliged by the European Medicines Agency (EMA) to perform tapering studies, as witnessed by, for example, the data from the ABILITY 3 study.

A final consideration is the open-label strategy. A general disadvantage of open-label studies is the risk of expectation bias (placebo/nocebo effect). However, since this trial studies a disease activity-guided strategy, which can result in flares rather than response to treatment, expectation bias favouring tapering is not likely. Since this is a non-inferiority design, expectation bias would rather result in inferiority of the tapering group, resulting in a more conservative estimation of the effect. Open-label tapering studies in RA indeed show that successful dose optimisation is achieved in a smaller proportion of patients compared to similar blinded studies [9]. This may in part be due to the patient’s fear of decreasing their dose and experiencing a flare, leading to the nocebo effect and incorrect attribution of complaints to the dose optimisation. However, since in clinical practice tapering is not blinded either, open-label studies may more realistically reflect chances of success. Success of tapering depends not only on pharmacological factors, but as much on psychological factors, such as thorough information from the physician, or inter-patient variability in fear of flares. Therefore, open-label tapering results in an approximation of the effect in daily clinical practice but possibly an underestimation of the full pharmacological possibility.

### Trial status

The trial started with recruitment on 9 January 2018 and will be completed by approximately 29 January 2021. Protocol version 1.4, 01-09-2019.

## Supplementary information

**Additional file 1.** SPIRIT 2013 Checklist: Recommended items to address in a clinical trial protocol and related documents.

## Data Availability

The data collected during the study are available to researchers who provide a methodologically sound proposal to achieve aims in the approved proposal or for individual participant data meta-analysis. To gain access, data requestors will need to sign a data access agreement.

## Abbreviations

ACR: American College of Rheumatology
ANCA: Anti-neutrophil cystoplasmic antibody
AS: Ankylosing spondylitis
ASAS: Assessment of SpondyloArthritis International Society
ASAS-HI: Assessment of SpondyloArthritis International Society Health Index
ASDAS: Ankylosing Spondylitis Disease Activity Score
AUC: Area under the curve
AVG: Algemene Verordening Gegevensbescherming
axSpA: Axial spondyloarthritis
BASFI: Bath Ankylosing Spondylitis Functional Index
bDMARD: Biological disease-modifying anti-rheumatic drug
BSA: Body surface area
CASPAR: Classification Criteria for Psoriatic Arthritis
CMO: Commissie Mensgebonden Onderzoek
CRP: C-reactive protein
csDMARD: Conventional synthetic disease modifying anti-rheumatic drug
CTLA4-Ig: Cytotoxic T-Lymphocyte Associated Protein 4-Immunoglobulin
DAPSA: Disease activity in psoriatic arthritis
DAS28-CRP: Disease Activity Score 28-C-reactive protein
DDD: Defined daily dose
DMARD: Disease-modifying anti-rheumatic drug
DRESS: Dose reduction strategy study
DRESS-PS: Dose reduction strategy study of TNF inhibitors in psoriatic arthritis and axial spondyloarthritis
DSMB: Data Safety Monitoring Board
EMA: European Medicines Agency
EULAR: European League Against Rheumatism
EuroQoL-5D-3 L: Health status
GCP: Good Clinical Practice
GDPR: General Data Protection Regulation
GPA: Granulomatosis with polyangiitis
HAQ-DI: Health Assessment Questionnaire Disability Index
IBD: Inflammatory bowel disease
ITT: Intention to treat
JAKi: Janus Kinase inhibitor
LDA: Low disease activity
MAR: Missing at random
mBSA: Modified body surface area
MCAR: Missing completely at random
MCIW: Minimal clinically important worsening
MDA: Minimal disease activity
mNY: Modified New York criteria
MPO: Myeloperoxidase
MSASSS: Modified Stoke Ankylosing Spondylitis Spine Score
NI: Non-inferiority
NNH: Number needed to harm
NNT: Number needed to treat
NraxSpA: Non-radiographic axial spondyloarthritis
NSAID: Non-steroidal anti-inflammatory drug
NTR: Nederlands Trial Register
OMERACT: Outcome measures in rheumatology
PASDAS: Psoriatic Arthritis Disease Activity Score
PDE-4i: Phosphodiesterase-4 inhibitor
PP: Per protocol
PR3: Proteinase 3
PsA: Psoriatic arthritis
QOL: Quality of life
RA: Rheumatoid arthritis
Radboudumc: Radboud University Medical Centre
RCT: Randomised Controlled Trial
RF-: Rheumatoid factor negative
RF+: Rheumatoid factor positive
SD: Standard deviation
SF-12: Short Form-12 Health Survey
SI-joint: Sacroiliac joint
SpA: Spondyloarthritis
STRASS: Spacing of TNF-blocker injections in rheumatoid arthritis study
TNFi: Tumor necrosis factor inhibitors
tsDMARD: Targeted synthetic disease-modifying anti-rheumatic drug
VAS: Visual analogue scale

## References

[1] Ward MM, Deodhar A, Akl EA, Lui A, Ermann J, Gensler LS (2016). American College of Rheumatology/Spondylitis Association of America/Spondyloarthritis Research and Treatment Network 2015 Recommendations for the treatment of ankylosing spondylitis and nonradiographic axial spondyloarthritis. Arthritis Rheumatol..

[2] Gossec L, Smolen JS, Ramiro S, de Wit M, Cutolo M, Dougados M (2016). European League Against Rheumatism (EULAR) recommendations for the management of psoriatic arthritis with pharmacological therapies: 2015 update. Ann Rheum Dis.

[3] Smolen JS, Schols M, Braun J, Dougados M, FitzGerald O, Gladman DD (2018). Treating axial spondyloarthritis and peripheral spondyloarthritis, especially psoriatic arthritis, to target: 2017 update of recommendations by an international task force. Ann Rheum Dis.

[4] Ward Michael M., Deodhar Atul, Gensler Lianne S., Dubreuil Maureen, Yu David, Khan Muhammad Asim, Haroon Nigil, Borenstein David, Wang Runsheng, Biehl Ann, Fang Meika A., Louie Grant, Majithia Vikas, Ng Bernard, Bigham Rosemary, Pianin Michael, Shah Amit Aakash, Sullivan Nancy, Turgunbaev Marat, Oristaglio Jeff, Turner Amy, Maksymowych Walter P., Caplan Liron (2019). 2019 Update of the American College of Rheumatology/Spondylitis Association of America/Spondyloarthritis Research and Treatment Network Recommendations for the Treatment of Ankylosing Spondylitis and Nonradiographic Axial Spondyloarthritis. Arthritis & Rheumatology.

[5] van der Heijde D, Ramiro S, Landewe R, Baraliakos X, Van den Bosch F, Sepriano A (2017). 2016 update of the ASAS-EULAR management recommendations for axial spondyloarthritis. Ann Rheum Dis.

[6] Minozzi S, Bonovas S, Lytras T, Pecoraro V, Gonzalez-Lorenzo M, Bastiampillai AJ (2016). Risk of infections using anti-TNF agents in rheumatoid arthritis, psoriatic arthritis, and ankylosing spondylitis: a systematic review and meta-analysis. Expert Opin Drug Saf.

[7] Singh JA, Cameron C, Noorbaloochi S, Cullis T, Tucker M, Christensen R (2015). Risk of serious infection in biological treatment of patients with rheumatoid arthritis: a systematic review and meta-analysis. Lancet..

[8] Smolen JS, Landewe R, Bijlsma J, Burmester G, Chatzidionysiou K, Dougados M (2017). EULAR recommendations for the management of rheumatoid arthritis with synthetic and biological disease-modifying antirheumatic drugs: 2016 update. Ann Rheum Dis.

[9] Verhoef LM, van den Bemt BJ, van der Maas A, Vriezekolk JE, Hulscher ME, van den Hoogen FH (2019). Down-titration and discontinuation strategies of tumour necrosis factor-blocking agents for rheumatoid arthritis in patients with low disease activity. Cochrane Database Syst Rev.

[10] Vanier A, Mariette X, Tubach F, Fautrel B, Group SS (2017). Cost-effectiveness of TNF-blocker injection spacing for patients with established rheumatoid arthritis in remission: an economic evaluation from the spacing of TNF-blocker injections in rheumatoid arthritis trial. Value Health.

[11] den Broeder N, Bouman CAM, Kievit W, van Herwaarden N, van den Hoogen FHJ, van Vollenhoven RF (2019). Three-year cost-effectiveness analysis of the DRESS study: protocolised tapering is key. Ann Rheum Dis.

[12] Tran-Duy A, Ghiti Moghadam M, Oude Voshaar MAH, Vonkeman HE, Boonen A, Clarke P (2018). An economic evaluation of stopping versus continuing tumor necrosis factor inhibitor treatment in rheumatoid arthritis patients with disease remission or low disease activity: results from a pragmatic open-label trial. Arthritis Rheumatol..

[13] Coates LC, Navarro-Coy N, Brown SR, Brown S, McParland L, Collier H (2013). The TICOPA protocol (tight control of psoriatic arthritis): a randomised controlled trial to compare intensive management versus standard care in early psoriatic arthritis. BMC Musculoskelet Disord.

[14] Edwards CJ, Fautrel B, Schulze-Koops H, Huizinga TWJ, Kruger K (2017). Dosing down with biologic therapies: a systematic review and clinicians' perspective. Rheumatology (Oxford).

[15] Navarro-Compan V, Plasencia-Rodriguez C, de Miguel E, Balsa A, Martin-Mola E, Seoane-Mato D (2016). Anti-TNF discontinuation and tapering strategies in patients with axial spondyloarthritis: a systematic literature review. Rheumatology (Oxford).

[16] Cantini F, Niccoli L, Cassara E, Kaloudi O, Nannini C (2013). Duration of remission after halving of the etanercept dose in patients with ankylosing spondylitis: a randomized, prospective, long-term, follow-up study. Biologics..

[17] Gratacos J, Pontes C, Juanola X, Sanz J, Torres F, Avendano C (2019). Non-inferiority of dose reduction versus standard dosing of TNF-inhibitors in axial spondyloarthritis. Arthritis Res Ther.

[18] Landewe R, Sieper J, Mease P, Inman RD, Lambert RG, Deodhar A (2018). Efficacy and safety of continuing versus withdrawing adalimumab therapy in maintaining remission in patients with non-radiographic axial spondyloarthritis (ABILITY-3): a multicentre, randomised, double-blind study. Lancet..

[19] den Broeder AA, van Herwaarden N, van der Maas A, van den Hoogen FH, Bijlsma JW, van Vollenhoven RF (2013). Dose reduction strategy of subcutaneous TNF inhibitors in rheumatoid arthritis: design of a pragmatic randomised non inferiority trial, the DRESS study. BMC Musculoskelet Disord.

[20] van Herwaarden N, van der Maas A, Minten MJ, van den Hoogen FH, Kievit W, van Vollenhoven RF (2015). Disease activity guided dose reduction and withdrawal of adalimumab or etanercept compared with usual care in rheumatoid arthritis: open label, randomised controlled, non-inferiority trial. BMJ..

[21] van der Maas A, Lie E, Christensen R, Choy E, de Man YA, van Riel P (2013). Construct and criterion validity of several proposed DAS28-based rheumatoid arthritis flare criteria: an OMERACT cohort validation study. Ann Rheum Dis.

[22] Coates LC, Helliwell PS (2016). Treating to target in psoriatic arthritis: how to implement in clinical practice. Ann Rheum Dis.

[23] Atalay S, van den Reek J, van Vugt LJ, Otero ME, van de Kerkhof PCM, den Broeder AA (2017). Tight controlled dose reduction of biologics in psoriasis patients with low disease activity: a randomized pragmatic non-inferiority trial. BMC Dermatol.

[24] Clinical Trials: The European Union Clinical Trials Register. https://www.clinicaltrialsregister.eu/ctr-search/trial/2016-003321-42/NL. Accessed 20 August 2019.

[25] Tweehuysen L, van den Ende CH, Beeren FM, Been EM, van den Hoogen FH, den Broeder AA (2017). Little evidence for usefulness of biomarkers for predicting successful dose reduction or discontinuation of a biologic agent in rheumatoid arthritis: a systematic review. Arthritis Rheumatol.

[26] Salaffi F, Ciapetti A, Carotti M, Gasparini S, Gutierrez M (2014). Disease activity in psoriatic arthritis: comparison of the discriminative capacity and construct validity of six composite indices in a real world. Biomed Res Int.

[27] Perruccio AV, Got M, Li S, Ye Y, Gladman DD, Chandran V. Treating psoriatic arthritis to target: defining Psoriatic Arthritis Disease Activity Score (PASDAS) that reflects state of minimal disease activity (MDA). J Rheumatol. 2019. 10.3899/jrheum.181472. 10.3899/jrheum.181472 31203221

[28] Helliwell PS, FitzGerald O, Fransen J, Gladman DD, Kreuger GG, Callis-Duffin K (2013). The development of candidate composite disease activity and responder indices for psoriatic arthritis (GRACE project). Ann Rheum Dis.

[29] Coates LC, Helliwell PS (2016). Defining low disease activity states in psoriatic arthritis using novel composite disease instruments. J Rheumatol.

[30] Helliwell PS, FitzGerald O, Fransen J (2014). Composite disease activity and responder indices for psoriatic arthritis: a report from the GRAPPA 2013 meeting on development of cutoffs for both disease activity states and response. J Rheumatol.

[31] Molto A, Gossec L, Meghnathi B, Landewe RBM, van der Heijde D, Atagunduz P (2018). An Assessment in SpondyloArthritis International Society (ASAS)-endorsed definition of clinically important worsening in axial spondyloarthritis based on ASDAS. Ann Rheum Dis.

[32] Machado PM, Landewe R, Heijde DV (2018). Assessment of SpondyloArthritis international S. Ankylosing Spondylitis Disease Activity Score (ASDAS): 2018 update of the nomenclature for disease activity states. Ann Rheum Dis.

[33] Piaggio G, Elbourne DR, Pocock SJ, Evans SJ, Altman DG, Group C (2012). Reporting of noninferiority and equivalence randomized trials: extension of the CONSORT 2010 statement. JAMA..

[34] Rehal S, Morris TP, Fielding K, Carpenter JR, Phillips PP (2016). Non-inferiority trials: are they inferior? A systematic review of reporting in major medical journals. BMJ Open.

[35] Fautrel B, Pham T, Alfaiate T, Gandjbakhch F, Foltz V, Morel J (2016). Step-down strategy of spacing TNF-blocker injections for established rheumatoid arthritis in remission: results of the multicentre non-inferiority randomised open-label controlled trial (STRASS: Spacing of TNF-blocker injections in rheumatoid arthritis study). Ann Rheum Dis.

[36] Campbell G. Guidance for the Use of Bayesian Statistics in Medical Device Clinical Trials. 2010. https://www.fda.gov/regulatory-information/search-fda-guidance-documents/guidance-use-bayesian-statistics-medical-device-clinical-trials/. Accessed 20 Aug 2019.

[37] Sterne JA, White IR, Carlin JB, Spratt M, Royston P, Kenward MG (2009). Multiple imputation for missing data in epidemiological and clinical research: potential and pitfalls. BMJ..

[38] Moons KG, Donders RA, Stijnen T, Harrell FE (2006). Using the outcome for imputation of missing predictor values was preferred. J Clin Epidemiol.

[39] Goekoop-Ruiterman YP, de Vries-Bouwstra JK, Allaart CF, van Zeben D, Kerstens PJ, Hazes JM (2008). Clinical and radiographic outcomes of four different treatment strategies in patients with early rheumatoid arthritis (the BeSt study): a randomized, controlled trial. Arthritis Rheum.

[40] Heimans L, Akdemir G, Boer KV, Goekoop-Ruiterman YP, Molenaar ET, van Groenendael JH (2016). Two-year results of disease activity score (DAS)-remission-steered treatment strategies aiming at drug-free remission in early arthritis patients (the IMPROVED-study). Arthritis Res Ther..

[41] Mottonen T, Hannonen P, Leirisalo-Repo M, Nissila M, Kautiainen H, Korpela M (1999). Comparison of combination therapy with single-drug therapy in early rheumatoid arthritis: a randomised trial. FIN-RACo trial group. Lancet..

[42] Verschueren P, De Cock D, Corluy L, Joos R, Langenaken C, Taelman V (2015). Methotrexate in combination with other DMARDs is not superior to methotrexate alone for remission induction with moderate-to-high-dose glucocorticoid bridging in early rheumatoid arthritis after 16 weeks of treatment: the CareRA trial. Ann Rheum Dis.

[43] de Jong PH, Hazes JM, Han HK, Huisman M, van Zeben D, van der Lubbe PA (2014). Randomised comparison of initial triple DMARD therapy with methotrexate monotherapy in combination with low-dose glucocorticoid bridging therapy; 1-year data of the tREACH trial. Ann Rheum Dis.

[44] van Lonkhuyzen L. Prijzenoorlog rond bestverkochte medicijn tegen reuma. NRC. 2018. https://www.nrc.nl/nieuws/2018/12/05/prijzenoorlog-rond-bestverkochte-medicijn-tegen-reuma-humira-a3059663/. Accessed 20 Aug 2019.

[45] Medicijnkosten.Zorginstituut Nederland.2019. https://www.medicijnkosten.nl/. Accessed 20 August 2019.

